# Viscoelastic Hemostatic Testing as a Diagnostic Tool for Trauma-Induced Coagulopathy with a Focus on Arabian Gulf Countries: A Narrative Review

**DOI:** 10.3390/jcm15208048

**Published:** 2026-10-16

**Authors:** Khaled Ahmed Yassen, Klaus Görlinger, Lama Alzimami, Noor Salman Alswilem, Amani Almutairi, Mubarak Alsagheer, Hashim Alwayel, Ali Mousa Alkhalaf, Nour Hassan Aljamaan, Noor Alqurqush, Amnah Ali Alkhawajah, Ridha Alhussain, Sherif Saleh, Imtiyaz Ahmad Khan

**Affiliations:** 1 Anesthesia Unit, Surgery Department, King Faisal University, Hofuf 31982, Alahsa, Saudi Arabia; 2 Anesthesia Department, University of Duisburg, 45147 Essen, Germany; 3 Tem Innovations, 81829 Munich, Germany; kgoerlinger@werfen.com; 4 College of Medicine, King Faisal University, Hofuf 31982, Alahsa, Saudi Arabia; lamazaid1422@gmail.com (L.A.); 223042178@student.kfu.edu.sa (N.S.A.); itsamani19@gmail.com (A.A.); 221435805@student.kfu.edu.sa (M.A.); hashimalwail@gmail.com (H.A.); alimousa1423@gmail.com (A.M.A.); aljmannoor@gmail.com (N.H.A.); noor.aq@hotmail.com (N.A.); amnahalkhawajah@gmail.com (A.A.A.); ridhamedical@gmail.com (R.A.); 5 Surgery Department, College of Medicine, King Faisal University, Hofuf 31982, Alahsa, Saudi Arabia; ssaleh@kfu.edu.sa (S.S.); iakhan@kfu.edu.sa (I.A.K.)

**Keywords:** trauma-induced coagulopathy, viscoelastic hemostatic testing, thromboelastography, rotational thromboelastometry, hemostatic resuscitation, massive transfusion, fibrinolysis

## Abstract

Trauma-induced coagulopathy (TIC) at admission to the emergency department is associated with severe bleeding, massive transfusion, increased morbidity, and mortality. There are significant regional variations in the accessibility and acceptance of point-of-care technology, such as viscoelastic hemostatic testing (VET), for coagulation monitoring and bleeding management in the Arabian Gulf council countries (GCC). Notable regional differences among these countries are mainly due to variation in the awareness of the benefits of VET-guided bleeding management using rotational thromboelastometry (ROTEM) or thromboelastography (TEG), compared with a transfusion ratio-based approach using shock packs, rather than for economic reasons. This review highlights the superiority of VET-guided bleeding management in severe trauma over conventional coagulation tests (CCTs) for early TIC detection and the importance of monitoring hemostatic disorders, whether hypocoagulability or hypercoagulability. VET consistently outperforms traditional coagulation tests in detecting early coagulation abnormalities and predicting massive transfusion and mortality in major trauma. Besides hyperfibrinolysis, fibrinogen deficiency often represents the first issue in TIC. Recent meta-analyses consistently demonstrated a reduction in mortality by implementing VET-guided bleeding management algorithms in severe trauma. Several studies demonstrated that VETs are valuable tools for massive transfusion and mortality prediction in bleeding patients with severe trauma. There is a notable variation in VET adoption and practice across trauma settings in the Arab Gulf. GCC-specific primary evidence is limited, with the Qatar cohort representing the largest directly identified regional study.

## 1. Introduction

Major trauma (MT) is one of the world’s main causes of morbidity and mortality [1]. The Gulf Cooperation Council (GCC) has made the development of trauma systems a regional priority, although the pace of development varies across the countries in the region [2]. Trauma-induced coagulopathy (TIC) is one of the major consequences of road traffic accidents with major blood loss [1,3]. The laboratory conventional coagulation tests (CCTs), such as prothrombin time (PT) and activated partial thromboplastin time (aPTT), have failed in timely detection of trauma-induced coagulopathy in this acute setting due to their long turnaround times and inability to capture the dynamic interplay among clot formation, stability, and disintegration [4,5,6,7,8,9]. Viscoelastic testing (VET) has emerged as an alternative and crucial instrument for the immediate assessment of the coagulation status [10,11,12,13,14,15,16,17]. However, the availability and acceptance of the point-of-care technologies for bedside coagulation monitoring remain challenging in many parts of the world.

## 2. Methods

### 2.1. Review Design and Search Strategy

A structured narrative review was undertaken to examine the role of viscoelastic hemostatic testing (VET), principally thromboelastography (TEG) and rotational thromboelastometry (ROTEM), in the assessment and management of trauma-induced coagulopathy (TIC), with particular consideration of its application in Gulf Cooperation Council trauma systems. The review was informed by SANRA recommendations for narrative reviews [18]. Study identification and selection were documented using a PRISMA 2020-style flow diagram [19], and the search was reported in line with PRISMA-S [20].

PubMed/MEDLINE was searched on 22 August 2026 for English-language publications from 1 January 2010 to 22 August 2026. The search combined terms for trauma/injury with terms for VET, including TEG, ROTEM, thromboelastography, thromboelastometry, and viscoelastic testing. No restriction for geographic region, outcome, or study design was applied at retrieval. The complete search strategy is provided in the main text. The search retrieved 1552 records.

### 2.2. Eligibility and Study Selection

Original studies of adult human trauma patients were eligible when VET was used substantively for diagnosis or phenotyping of coagulopathy, assessment of fibrinolysis or platelet/fibrinogen abnormalities, prognostic evaluation, prediction of transfusion or clinical outcomes, or guidance of hemostatic treatment. Randomized and non-randomized intervention studies and diagnostic, prognostic, prospective, and retrospective observational studies were considered. Pediatric-only, non-trauma, non-human/in vitro, and non-primary publications were excluded, as were studies in which VET was incidental to the main research question.

Titles/abstracts and subsequently eligible complete articles were screened independently by three reviewers, with disagreements resolved by consensus. Complete article text was required for formal eligibility; reports that could not be retrieved were recorded as not retrieved and were not classified from the abstract alone. Companion publications arising from the same trial or cohort were linked into study families using study center, recruitment period, population and sample characteristics, intervention or VET protocol, trial/registry identity, and author overlap. The final evidence set comprised 261 eligible reports representing 233 unique study families.

### 2.3. Data Extraction and Methodological Appraisal

Data have been extracted using a standardized framework by one reviewer and checked by two additional reviewers. Extracted information included study design and setting, population, sample size, VET platform and assay, timing of measurement, VET parameters or thresholds, comparator tests, hemostatic interventions, transfusion requirements, and clinically relevant outcomes.

Methodological appraisal was selected according to the principal study question. Randomized trials were assessed with RoB 2 [21]; diagnostic-accuracy studies with QUADAS-3 [22]; prognostic studies with QUIPS [23]; observational cohort studies with the revised JBI cohort tool [24]; and non-randomized or quasi-experimental intervention studies with the revised JBI quasi-experimental tool [25]. Risk-of-bias judgments informed interpretation but were not used as an eligibility criterion.

### 2.4. Evidence Synthesis

Because of substantial heterogeneity in trauma populations, VET platforms and assays, sampling times, diagnostic thresholds, treatment algorithms, and reported outcomes, the evidence was reported narratively, and no meta-analysis was performed. Findings were grouped by the clinical role of VET, including diagnostic performance, coagulation and fibrinolytic phenotypes, prediction of transfusion and clinical outcomes, VET-guided hemostatic management, traumatic brain injury and platelet dysfunction, and post-traumatic hypercoagulability or thromboembolic risk.

### 2.5. Limitations of the Methodology

Inherent limitations of a narrative review include potential selection bias and a lack of quantitative synthesis. Variability in study design and reporting may limit direct comparability [26,27].

### 2.6. Rotational Thromboelastometry (ROTEM)

ROTEM (Tem Innovations, Munich, Germany) is one of the point-of-care tools for VET and assesses the kinetics and viscoelastic characteristics of the entire coagulation process in vitro for both intrinsic and extrinsic pathways [28]. Extrinsic coagulation pathway (EXTEM), intrinsic coagulation pathway (INTEM), fibrin contribution to clot firmness (FIBTEM), antifibrinolytic pharmaceutical effect (APTEM), and heparinase activity on intrinsically activated blood samples (HEPTEM) are the five main tests used with ROTEM [29].

**Figure 1:**
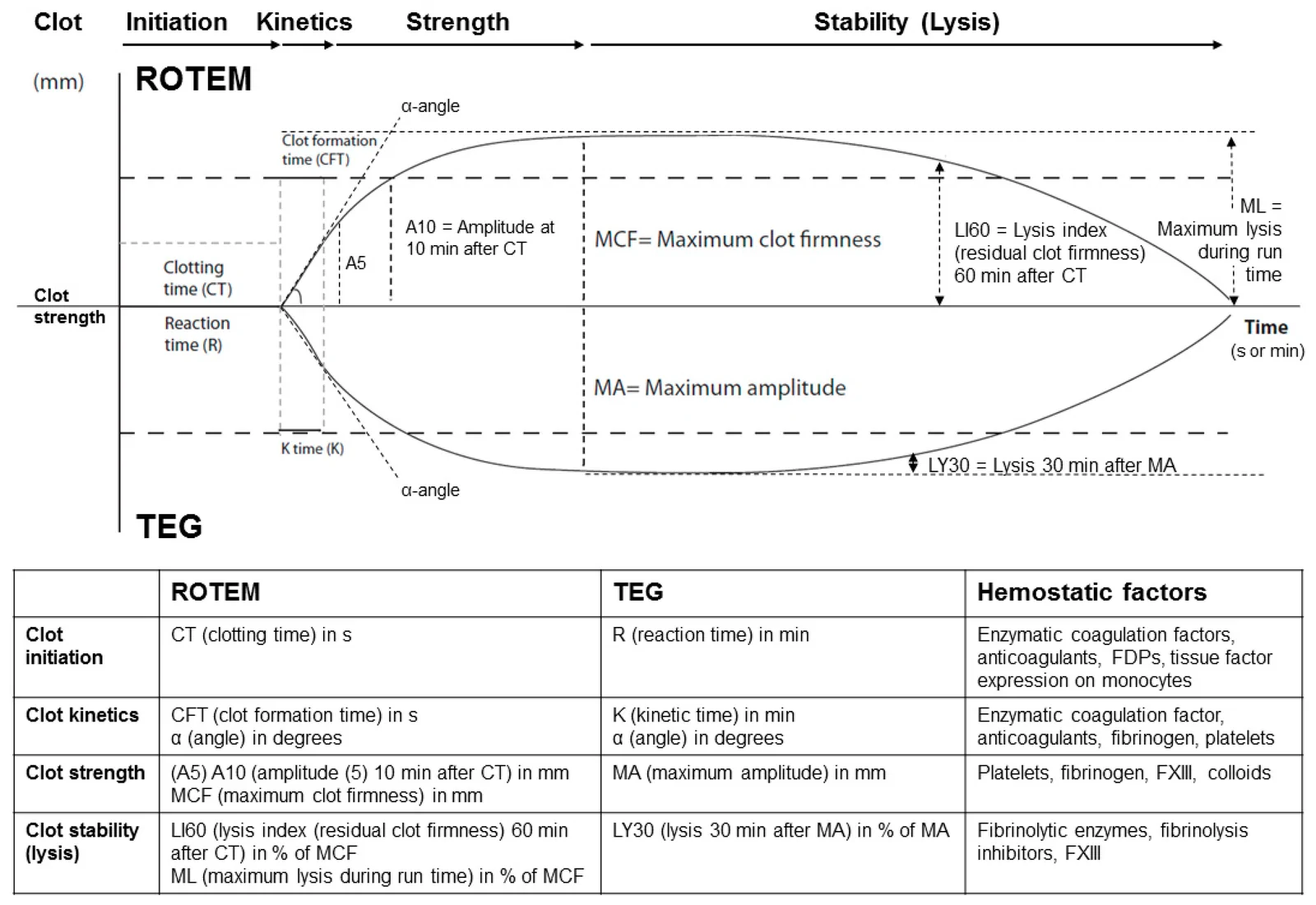
ROTEM and TEG parameters and indices. ROTEM: CT: coagulation time in seconds (time from test start to 2 mm clot firmness). CFT: clot formation time in seconds (time from 2 to 20 mm clot firmness). A5: clot firmness amplitude 5 min after CT in mm. A10: clot firmness amplitude 10 min after CT in mm. FDPs: fibrinogen degradation products, F XIII: coagulation factor XIII. LI30: lysis index 30 min after CT in % (residual clot firmness in percentage of MCF). LI45: lysis index 45 min after CT in %, LI60: lysis index 60 min after CT in %, LOT: lysis onset time in seconds (time from CT to 15% fibrinolysis = 85% residual clot firmness compared to MCF), and LT: lysis time in seconds (time from CT to 90% fibrinolysis = 10% residual clot firmness compared to MCF). TEG: R: reaction time in minutes (time from test start to 2 mm clot firmness). K-time: kinetic time in minutes (time from 2 to 20 mm clot firmness). Alpha angle: slope in degree. MA: maximum amplitude in mm. LY30: lysis at 30 min after MA in % of MA. Courtesy of Klaus Görlinger, Munich, Germany.

Like prothrombin time (PT), EXTEM monitors tissue factor activation and offers information on the extrinsic coagulation pathway [30,31]. Interference of heparin-like effects (up to 5 U/mL) are eliminated by polybrene. Similar to activated partial prothrombin time (aPTT), INTEM assesses contact activation and the intrinsic coagulation system [32,33]. HEPTEM eliminates heparin-like effects through heparinase (up to 7 U/mL) and thereby allows for differentiation of heparin, protamine, and the deficiency of clotting factor of the intrinsic pathway. By employing cytochalasin D, an actin polymerization inhibitor, and tirofiban, an GPIIbIIIa-receptor antagonist, to stop platelet involvement in clot formation, FIBTEM solely evaluates the effects of fibrin (Figure 1) [28,34,35,36]. Recent prospective longitudinal data by Hosala et al. indicated a substantial link between Clauss fibrinogen and FIBTEM MCF (r = 0.811). Strong methodological agreement between Clauss fibrinogen concentration and FIBTEM-derived clot firmness measures under dynamically changing perioperative settings is confirmed. Important prospective information on perioperative hemostasis monitoring in major non-cardiac surgery utilizing both ROTEM and traditional laboratory tests is provided by the abovementioned Hosala et al. trial. The methodological agreement between viscoelastic and traditional fibrinogen testing is supported by their results [37].

Key ROTEM parameters are clotting time (CT), amplitude 5 min after clotting time (A5), amplitude 10 min after clotting time (A10), maximum lysis (ML), and clot lysis index 60 min after clotting time (LI60) [29,38]. The threshold parameters for ROTEM are described in the prospective observational multicenter European study by Baksaas-Aasen et al. (2019) and others [29,39,40,41].

### 2.7. Thromboelastography (TEG)

In TEG (Haemonetics Corp., Braintree, MA, USA), Kaolin-TEG uses kaolin to activate the intrinsic pathway. Heparinase-TEG is activated by kaolin, too, but eliminates a heparin effect by heparinase. Rapid-TEG speeds up activation by using tissue factor in addition to kaolin. TEG Functional Fibrinogen (TEG-FF) uses the GPIIbIIIa-receptor antagonist abciximab for platelet inactivation [42,43]. However, it must be considered that abciximab alone results in an incomplete platelet inactivation with a significant “platelet noise” in the TEG-FF results [43]. Key TEG parameters are reaction time (R), kinetic time (K), maximum amplitude (MA), and lysis index 30 (lysis in % of MA 30 min after MA) [16,17]. The threshold parameters for TEG to guide bleeding management in severe trauma are described in the prospective observational multicenter European study by Baksaas-Aasen et al. (2019) too [39,44].

### 2.8. Trauma-Induced Coagulopathy (TIC)

TIC is triggered by severe tissue trauma and shock and is mainly caused by protein C-activation, hyperfibrinolysis, hypofibrinogenemia, platelet dysfunction, and endothelial dysfunction [8,14,15,45,46,47,48]. Acidosis, hypothermia, or hemodilution can contribute to TIC. Ostrowski et al. (n = 404) reported that elevated adrenaline and endothelial cell injury were independently associated with hypocoagulability and hyperfibrinolysis, which contribute to TIC [49].

## 3. Results

### 3.1. Study Selection

**Figure 2:**
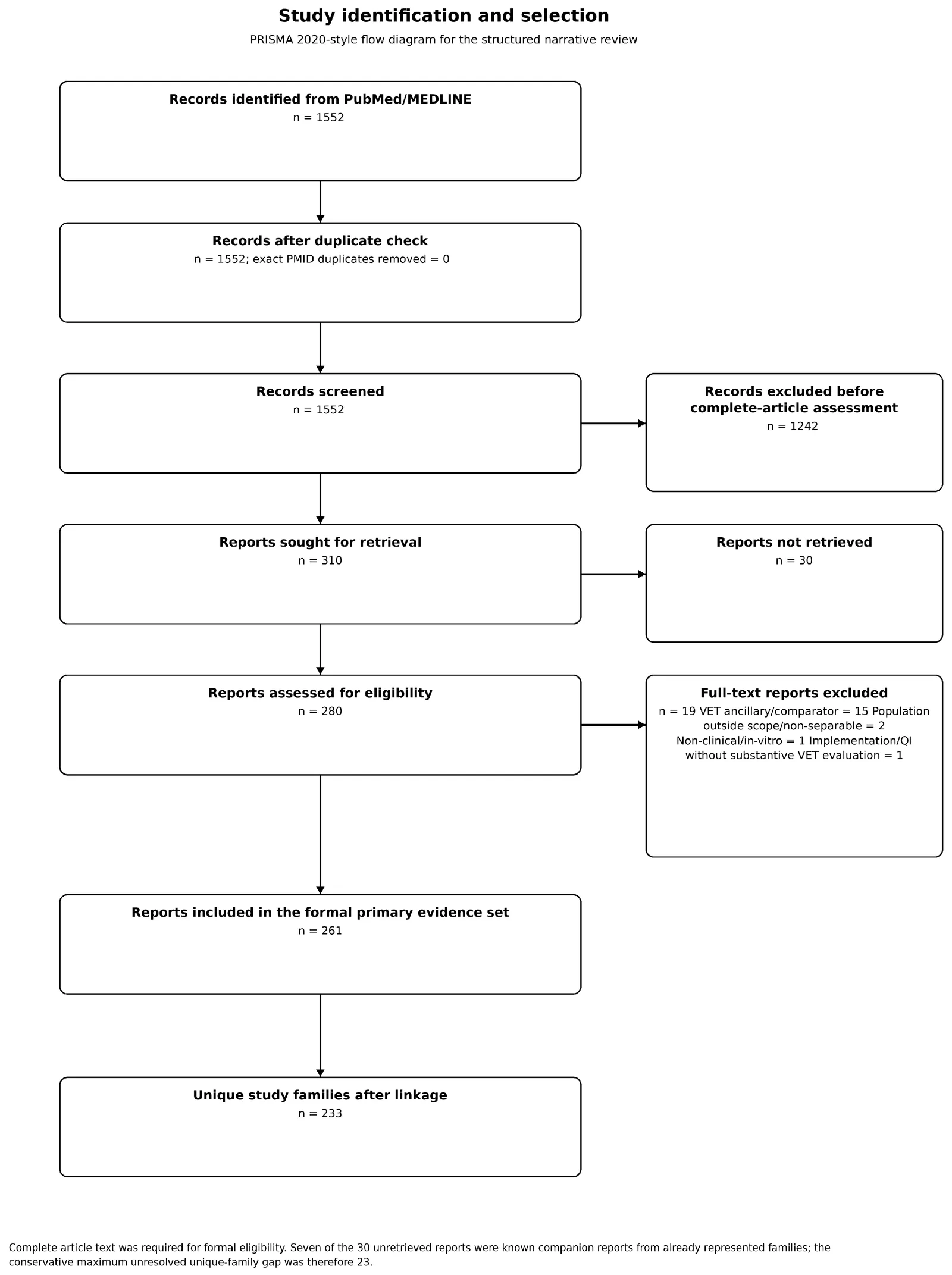
PRISMA 2020 flow chart for study selection.

The PubMed/MEDLINE search yielded 1552 records. After title/abstract screening, 310 reports were sought for complete article assessment. Thirty reports could not be retrieved, leaving 280 complete articles for eligibility assessment. Nineteen were excluded at this stage: VET was ancillary or used only as a comparator in 15 reports, two had an ineligible or non-separable population, one was non-clinical/in vitro, and one was an implementation/quality-improvement report without substantive patient-level VET evaluation. The final evidence set comprised 261 eligible reports representing 233 unique study families. The selection process is shown in Figure 2. Table 1 and Table 2 explain the detailed search results.

### 3.2. Characteristics of the Evidence Base

The 261 included reports were published between 2010 and 2026. Publication increased over time: 51 (19.5%) reports were published in 2010–2014, 90 (34.5%) in 2015–2019, and 120 (46.0%) in 2020–2026. Prognostic or prediction studies formed the largest methodological group (109/261, 41.8%), followed by diagnostic or assay-performance studies (60/261, 23.0%) and observational phenotype/association studies (52/261, 19.9%). Intervention-focused evidence included 25 non-randomized or quasi-experimental reports (9.6%) and 15 randomized intervention reports (5.7%). A numeric sample size was reliably available for 186 reports; among these, the median sample size was 134 (IQR 57.75–401; range 10–7461).

TEG was explicitly represented in 161 reports (61.7%) and ROTEM in 105 (40.2%); some reports used both platforms. Among named ROTEM assays, FIBTEM appeared in 45 reports, EXTEM in 38, and INTEM in 12. Coagulation/coagulopathy was the most frequently extracted clinical domain (215 reports), while diagnostic/prognostic performance and transfusion/blood product outcomes each appeared in 152 reports. Mortality/survival was reported in 151, hemostatic management in 135, fibrinogen in 103, platelet function in 98, fibrinolysis in 74, and thromboembolism in 22.

Seventeen confirmed multi-report study families accounted for 45 reports, whereas 216 reports were singletons. Collapsing companion publications therefore removed 28 report-level repetitions from the study-level denominator, yielding 233 unique study families.

### 3.3. Methodological Appraisal

On the project-level harmonized appraisal, 221 of 261 reports (84.7%) were classified as having moderate methodological concern and 40 (15.3%) as having high concern. At study-family level, 194 of 233 families (83.3%) were classified as moderate concern and 39 (16.7%) as high concern. The recurrent limitations were residual confounding in prognostic and observational analyses, selection and comparability issues in retrospective cohorts, non-randomized allocation or historical comparators in quasi-experimental studies, and threshold derivation or spectrum/transportability concerns in diagnostic studies. In randomized trauma trials, open-label emergency-care pathways commonly introduced potential deviations from intended management.

### 3.4. Diagnostic and Prognostic Evidence

Diagnostic and assay-performance studies evaluated VET for early recognition of TIC, detection of hypofibrinogenemia and fibrinolytic abnormalities, comparison of TEG and ROTEM platforms, and derivation of treatment-relevant thresholds. Large multicenter work developed data-driven ROTEM and TEG algorithms for trauma hemorrhage [38], while other studies derived ROTEM thresholds for massive transfusion [50], compared TEG 6s with TEG 5000 [51], and assessed VET-based fibrinogen measurements against plasma fibrinogen [52]. Because assays, reference constructs, sampling times, and thresholds differed substantially, diagnostic estimates were not pooled.

Prognostic/prediction questions were the largest evidence category. VET variables and phenotypes were evaluated against massive transfusion, mortality, thromboembolic events, and other adverse outcomes. Representative studies included rapid tPA-challenge TEG for prediction of massive transfusion [53], plasmin TEG for massive transfusion, mortality, and hyperfibrinolysis [54], admission rapid-TEG for subsequent pulmonary embolism [55], and serial thromboelastometry for the transition toward post-injury hypercoagulability [56]. The effect estimates were not pooled because prognostic definitions, VET thresholds, timing, resuscitation exposure, and modeling strategies were heterogeneous.

### 3.5. VET-Guided Hemostatic Management and Randomized Evidence

Forty reports had an intervention-focused dominant question, including 15 randomized and 25 non-randomized or quasi-experimental reports. The major randomized studies addressed different clinical strategies and did not show a uniform treatment effect.

In a pragmatic single-center trial of 111 patients, TEG-guided massive transfusion management reduced 28-day mortality from 36.4% with conventional coagulation-assay guidance to 19.6% (*p* = 0.049); early plasma and platelet use was also lower, whereas red-cell use was similar [57]. By contrast, the multicenter iTACTIC trial included 396 patients and found no overall difference in the proportion of patients alive and free of massive transfusion at 24 h (67% with VHA vs. 64% with conventional coagulation tests; OR 1.15, 95% CI 0.76–1.73) or in 28-day mortality (25% vs. 28%; OR 0.84, 95% CI 0.54–1.31) [58].

Fibrinogen-directed trials addressed product choice and speed of replacement rather than the same primary outcome. RETIC randomized 100 patients with ROTEM-defined coagulopathy to fresh frozen plasma or coagulation factor concentrates. Rescue therapy was required in 52% versus 4%, respectively (OR 25.34, 95% CI 5.47–240.03; *p* < 0.0001), and massive transfusion in 30% versus 12% (*p* = 0.042); multiple organ failure occurred in 66% versus 50% (*p* = 0.15) [59]. Table 3. Represents the principal randomized evidence relevant to VET-guided hemostatic management [57,58,59,60].

In the FEISTY pilot trial, 100 patients were randomized and 62 hypofibrinogenemic patients received fibrinogen replacement. Delivery was faster with fibrinogen concentrate than cryoprecipitate (median 29 vs. 60 min; *p* = 0.0001), while transfusion requirements and thromboembolic complications were similar [60].

### 3.6. Fibrinogen, Fibrinolysis, Platelet Function, and Thromboembolic Phenotypes

The evidence base extended beyond early hypocoagulability. Fibrinogen-related questions appeared in 103 reports and fibrinolysis in 74. Studies examined both hyperfibrinolysis and fibrinolysis shutdown, including the limited sensitivity of standard thromboelastometry for some fibrinolytic states [61] and temporal transitions toward late hypofibrinolysis associated with adverse outcomes [62]. Platelet function was addressed in 98 reports, including comparisons of TEG platelet mapping with other platelet function assays [63] and traumatic brain injury-related platelet dysfunction [64]. Twenty-two reports evaluated thromboembolic outcomes or post-traumatic hypercoagulability. Because phenotype definitions varied by platform, reagent, and sampling time, phenotype frequencies were not combined across studies.

### 3.7. GCC-Specific Evidence

Directly identifiable GCC primary evidence was limited. A contemporary cohort from Qatar included 1488 adult trauma patients undergoing admission ROTEM; 376 (25.3%) had ROTEM abnormalities. Abnormal coagulation profiles and hypofibrinogenemia were associated with greater injury severity, transfusion requirement, and mortality, and FIBTEM measurements were evaluated in relation to plasma fibrinogen [48]. This was the largest directly identified GCC-specific cohort in the formal evidence set.

## 4. Discussion

Retrospective and observational data represent the bulk of the published data retrieved. The number of RCTs on the effect of VET in trauma is limited. Most studies back up the use of ROTEM or TEG in the emergency room to diagnose or exclude TIC and guide blood component transfusions or the use of coagulation factor concentrates [17]. When VETs are used, blood components and coagulation factor concentrates are judiciously transfused [17,65,66,67].

This review revealed four main points: The superiority of ROTEM over CCTs for early TIC detection, the increasing evidence regarding mortality benefit, the importance of fibrinogen deficiency as an early issue in TIC, and the significant variation in VET adoption and practice across trauma settings in the Arab Gulf region.

### 4.1. Diagnostic Performance of VET for Early TIC Detection

Several studies reported that VET identified clinically significant coagulation abnormalities earlier and with greater sensitivity than CCT, highlighting the limitations of conventional testing in acute trauma settings. Table 4 presents details of the studies supporting the above findings [57,68,69,70,71]. VETs can quickly identify clinically significant anomalies within minutes and at different degrees [72]. Kashuk et al. described in 2012 how patients with major trauma received significantly fewer blood units (*p* = 0.048) when compared with the period before TEG implementation [73]. According to a retrospective cohort study from Qatar, 25% of trauma patients had abnormal ROTEM values upon admission, while only 5% had abnormal PT readings. VETs have a higher sensitivity for early coagulopathy diagnosis [48]. Cucher and his colleagues performed a retrospective analysis of 772 patients with traumatic brain injury (TBI) between 2014 and 2017. They described that CCTs underestimate TBI-associated coagulopathy compared to TEG [74]. Similar results have been reported for ROTEM by Rimaitis et al. [75,76].

### 4.2. Diagnostic Performance of VET for Mortality Prediction in Severe Trauma

Kelly et al. (2018) reported in a retrospective analysis of 1146 trauma patients that ROTEM EXTEM A5, A10, and MCF predicted massive transfusion and in-hospital mortality [16,74,75]. Both, EXTEM A5 and A10 correlated well with EXTEM MCF, with Pearson coefficients of r = 0.92 and r = 0.96, respectively. The correlations of EXTEM A5, A10 and MCF with requirement for massive transfusion were all similarly high, with c-stats of 0.87, 0.89 and 0.90, respectively. The correlations with mortality were also similar but weaker, with c-stats of 0.67, 0.69 and 0.69, respectively. Using a previously validated cut-off of EXTEM A5 < 35 mm [15,16,74,75] to predict massive transfusion gave a sensitivity of 95%, specificity of 83%, positive predictive value of 9.3%, and negative predictive value of 100%. Using a value of A5 < 29 mm, for a pragmatic positive predictive value of 20%, gave a sensitivity of 67%, specificity of 95% and negative predictive value of 99%. Whether aiming for a high sensitivity or a strong predictive value, A5 was non-inferior to A10 and actually missed fewer cases needing massive transfusion. A5 has similar utility to both A10 and MCF as an early measure of clot firmness, and a low A5 value is strongly predictive of the need for massive transfusion.

Kildal et al. (2025) confirmed that a single ROTEM test at hospital admission is able to predict 30-day mortality in trauma using machine learning [89]. Here, ROTEM alone predicted 30-day mortality with an AUROC of 0.80, comparable to Revised Trauma Score (RTS) and New Injury Severity Score (NISS) (both 0.79) and superior to PT-INR (0.63) and base excess (0.58). When combined with ROTEM, specificity significantly improved across all trauma scores, with the greatest gains observed in RTS (0.23 to 0.62) and NISS (0.36 to 0.69) (all *p* < 0.001). Key predictive ROTEM variables included CT, CFT, and fibrinolysis indices (LI30, LI45, LI60).

Besides decreased early clot firmness amplitudes (EXTEM and FIBTEM A5 and A10), prolonged EXTEM CT > 90 s is associated with increased mortality too [31,36,38].

Furthermore, Wang et al. (2020) demonstrated that FIBTEM improved the sensitivity of hyperfibrinolysis detection in severe trauma patients [8,31,36,38]. The EXTEM HF (ML > 15% within 1 h; n = 37), FIBTEM HF (ML > 15% within 1 h; n = 132), and non-HF ML < 15% in 1 h in both tests; n = 233) groups had mortality rates of 81.1%, 22.3%, and 10.3%, respectively. The twofold difference in mortality rates between the FIBTEM HF and non-HF groups remained statistically significant after Bonferroni correction (*p* = 0.01). On multivariable Cox regression analysis, FIBTEM HF was independently associated with in-hospital mortality (adjusted hazard ratio 2.15, 95% CI 1.21–3.84, *p* = 0.009). Here, trauma patients with FIBTEM HF had significantly higher mortality rates than those without HF. FIBTEM is a valuable diagnostic method to improve HF detection’s sensitivity in trauma patients.

Accordingly, Chapman et al. (2015), Farrell et al. (2022), and Walsh et al. (2024) reported that severe hyperfibrinolysis in trauma, also called the “Death Diamond”, characterized by a rapid-TEG time to MA (TMA) ≤ 14 min and Time to Total Lysis after MA (TTL) < 30 min (or LY30 = 100%), was associated with poor survival and may be used as a futile indicator for stopping transfusion in severely bleeding trauma patients with hemorrhagic shock [6,90,91]. Mortality was 100% if the “Death Diamond” occurred in serial measurements [6,90,91].

Pezold et al. (2012) showed that the TEG clot strength parameter G can independently predict death early in the process of resuscitation when reviewing data from trauma patients between 2008 and 2010 [92]. Savage et al. 2017 demonstrated that mortality rates increased when TEG MA to R ratios decreased [93]. They linked this to fibrinogen depletion and recommended early cryoprecipitate infusion [93]. Smith et al. (2020) found in their retrospective study that a low TEG alpha angle and an elevated ROTEM APTEM CFT significantly predicted increased mortality whereas abnormal PT and aPTT did not [94]. Furthermore, Smith et al. (2022) reported that although males and females differed in TEG/ROTEM results, there was no difference in mortality [95].

### 4.3. Impact of VET Monitoring and Bleeding Management on Mortality in Severe Trauma

Three recent meta-analyses consistently demonstrated a significant reduction in mortality in the VET group [65,66,77]. Here, Bugaev et al. from the US Eastern Association for the Surgery of Trauma performed a meta-analysis in 2020 based on six studies and 1748 participants with a risk ratio (RR) for mortality [95% CI] of 0.75 [0.59; 0.95], a *p*-value of 0.02, and a heterogeneity of I^2^ of 0% for the TEG/ROTEM compared with the control group [64]. The significant reduction in mortality has been confirmed by a recent meta-analysis published by Mitra et al. in 2026 based on 29 studies and 11,006 participants with a RR [95% CI] of 0.789 [0.701; 0.888], a *p*-value of 0.0003, and a heterogeneity of I^2^ of 31% [77]. Again, the meta-analysis performed by the Cochrane group in 2026 demonstrated a significant reduction in mortality in studies using TEG/ROTEM-guided transfusion algorithms with a RR [95% CI] of 0.76 [0.63; 0.92] and a heterogeneity of I2 of 0% [65]. The Cochrane meta-analysis used 19 RCTs including 1865 participants from different clinical settings. In summary, all recent meta-analyses demonstrated a significant reduction in mortality in the TEG/ROTEM group compared with controls with a RR between 0.75 and 0.79. Accordingly, the implementation of VET-guided bleeding management in severe trauma is recommended in national and international guidelines with the level IA to IB [4,65,96]. National or regional trauma guidelines for the Gulf Cooperation Council countries are still missing—even if the development of a nationwide trauma system is a priority of the Vision 2030 of the Kingdom of Saudi Arabia [2,97,98,99].

In contrast to the abovementioned recent meta-analyses, older and outdated systematic reviews and meta-analyses found no significant difference in mortality in trauma [100,101]. Notably, the evidence for trauma is predominantly based on retrospective, observational studies and only two RCTs [13,57,58,69,78,79,80,81,82,83,84,85,86,87,88].

The two RCTs have been performed by Gonzalez et al. (2016) and Baksaas-Aasen et al. (2021) [57,58]. Gonzalez et al. [57] conducted a single-center pragmatic RCT including 111 trauma patients meeting the criteria for massive transfusion protocol (MTP) activation in an intent-to-treat analysis (TEG = 56, conventional coagulation assays (CCA) = 55). Survival in the TEG group was significantly higher than the CCA group (log-rank *p* = 0.032, Wilcoxon *p* = 0.027); 20 deaths in the CCA group (36.4%) compared with 11 in the TEG group (19.6%) (*p* = 0.049). Most deaths occurred within the first 6 h from arrival (21.8% CCA group vs. 7.1% TEG group) (*p* = 0.032). CCA patients required a similar number of red blood cell units as the TEG patients [CCA: 5.0 (2–11), TEG: 4.5 (2–8)] (*p* = 0.317), but more plasma units [CCA: 2.0 (0–4), TEG: 0.0 (0–3)] (*p* = 0.022) and more platelets units [CCA: 0.0 (0–1), TEG: 0.0 (0–0)] (*p* = 0.041) in the first 2 h of resuscitation.

Baksaas-Aasen et al. [58] performed a multicenter, RCT (iTACTIC) comparing outcomes in major trauma patients who received empiric massive hemorrhage protocols (MHPs), augmented by either VET or CCT-guided interventions. Of 396 patients in the intention-to-treat analysis, 201 were allocated to VET and 195 to CCT-guided therapy. At 24 h, there was no difference in the proportion of patients who were alive and free of massive transfusion (VET: 67%, CCT: 64%, OR 1.15, 95% CI 0.76–1.73); 28-day mortality was not different overall (VET: 25%, CCT: 28%, OR 0.84, 95% CI 0.54–1.31), nor were there differences in other secondary outcomes or serious adverse events. However, only 29% (102/355) of patients were coagulopathic (PT-ratio > 1.2) at baseline. In the pre-specified subgroups of coagulopathic patients OR [95% CI] for 28-day mortality was 0.56 (0.26; 1.24), *p* = 0.070, and for patients with severe TBI OR [95% CI] was 0.28 [0.10; 0.74], *p* = 0.016.

Table 4 represents eighteen studies demonstrating the superiority of viscoelastic hemostatic testing (VET)-guided bleeding management in severe trauma compared with CCT-guided bleeding management or the use of shock packs (transfusion-ratios) [13,57,58,65,66,69,77,78,79,80,81,82,83,84,85,86,87,88].

### 4.4. Hypo- and Hypercoagulability in Trauma

Coagulation profiles range from hypocoagulability to hypercoagulability 24 to 48 h following major trauma. Fibrinogen deficiency emerged as one of the earliest and most consistent abnormalities identified in TIC [14,102,103]. Higher rates of fibrinogen administration have been observed in the German Trauma Register DGU and other studies [59,85,104,105,106]. This is consistent with the pathophysiologic understanding that in traumatic hemorrhage, fibrinogen is frequently the first coagulation factor to decrease [48]. Fibrinogen is essential for the formation of clots and is thought to be a major factor in determining the strength of clots. A higher risk of bleeding during major surgery is often linked to hypofibrinogenemia. The utility of FIBTEM as a quick point-of-care estimation of fibrinogen-related clot strength is supported by Hosala et al.’s demonstration of a significant correlation between fibrinogen concentration according to Clauss and FIBTEM parameters (A10 and MCF). The use of viscoelastic testing as a quick functional evaluation of fibrinogen contribution to clot strength is supported by the strong agreement between fibrinogen concentration according to Clauss and FIBTEM parameters seen in the Hosala et al. study [37].

Some studies reported sex-related differences in coagulation profiles, with females showing a tendency toward hypercoagulability. Here, Coleman et al. (2019) and Schreiber et al. (2005) reported that females with severe injuries are more likely to present hypercoagulability (with increased TEG MA and less hyperfibrinolysis) compared with male patients [107,108].

Besides ISS and intraoperative transfusion rate, TEG (K-time, alpha-angle, MA) and ROTEM parameters (CFT, A10, MCF) provide excellent prediction (ROC AUC = 0.89) of postoperative venous thromboembolic events (VTEs) [109,110]. Besides prediction of VTEs, VET-guided bleeding management may prevent post-traumatic VTEs. Here, the incidence of venous thromboembolic events (VTEs) could be reduced in two trauma RCTs in the ROTEM/TEG group. In the RETIC RCT the incidence of VTEs was reduced from 18% to 8% (*p* = 0.22), in the iTACTIC RCT from 14% to 9% (*p* = 0.088), and in the combined dataset from 15% to 8% (*p* = 0.031) [57,58]. This is in line with a significant reduction in VTEs by VET-guided bleeding management in cardiac surgery (OR [95% CI], 0.44 [0.28; 0.70]; *p* = 0.0006) [111].

#### 4.4.1. Hyperfibrinolysis and Fibrinolysis Shutdown in TIC

Liberation of tPA in hemorrhagic shock with tissue hypoperfusion and activation of the protein C pathway can result in hyperfibrinolysis in severe trauma [112,113]. Furthermore, actin and other structural proteins released after cellular death during significant trauma can induce a hypercoagulable profile with decreased fibrinolysis in vitro [114].

Several authors demonstrated that fibrinolysis and fibrinolysis shutdown are associated with increased mortality [6,7,8,9,62,90]. Tranexamic acid (TXA) can be administered preemptively in patients with severe trauma, active bleeding and hemorrhagic shock within 90–180 min after the injury [115,116,117,118]. The prescription of TXA after 3 h following injury was based on an observation in the CRASH-2 study of increased mortality in patients receiving TXA after 3 h [115]. Later than 3 h after injury, TXA should only be given in cases of VET-based evidence of fibrinolysis in order to prevent prolonged pharmacologic fibrinolysis shutdown [119,120]. The main benefit of VET for trauma patients is to avoid withholding TXA from hyperfibrinolytic patients rather than to avoid administrating TXA to patients who are hypofibrinolytic or in fibrinolytic shutdown. In an RCT published by Harvin et al. (2015) including 1032 trauma patients with hyperfibrinolysis characterized by TEG LY30 ≥ 3%, logistic regression failed to find a difference in in-hospital mortality among those receiving TXA or not (OR [95% CI], 0.74 [0.38–1.40], *p* = 0.80 [121]. Moore et al. (2017) suggested to implement early goal-directed coagulation therapy to reduce mortality from hyper- and hypocoagulability after severe injury [122]. Mamczak et al. (2016) recommended the routine use of VET, since TEG/ROTEM use may result in transfusion ratios of blood products different from those of the current empiric 1:1:1 transfusion approach [123].

#### 4.4.2. Platelet Dysfunction in TIC

Platelet function declines markedly within the first 24 h after trauma, independent of platelet count, indicating an early qualitative dysfunction in addition to thrombocytopenia [124,125,126,127,128,129]. Numerous studies have examined the relationships between acidosis, coagulopathy, and injury severity [130,131]. According to a joint study by Cairo University in Egypt, Washington, Virginia Commonwealth, and Universities in Florida and Michigan in the United States, trauma in severe damage decreases platelet adhesion, aggregation, and contraction while preserving the ability to release alpha granules [132]. Durkin et al. (2025) found that 70% of adult trauma patients with significant hemorrhage exhibit platelet dysfunction when investigated with ROTEM, which supports the above findings [133]. Prior research by Solomon et al. (2011) confirmed these findings [134].

Coagulation function recovery varies and can differ from person to person. According to McCully et al. (2017), patients with venous thromboembolism as a result of severe trauma experienced delayed recovery of platelet function, namely with arachidonic acid-mediated platelet function recovery [135].

### 4.5. VET Availability Across ER Departments in the Arabian Gulf Region

The adoption of VET in trauma care varies significantly across the Arabian Gulf region. While countries such as Qatar have integrated VET into advanced trauma systems and hybrid emergency rooms, other regions continue to rely primarily on CCTs or 1:1:1 transfusion ratios [136,137,138,139,140].

Modern hybrid emergency rooms (ERs) with a multidisciplinary team approach and a range of treatment modalities were introduced recently in Qatar. These rooms maximized monitoring capabilities and reduced inter-hospital transfers of patients with severe injuries. VETs for early Patient Blood Management (PBM) are included in these hybrid rooms.

Mahmood et al. recently released a case study from Hamad Hospital in Qatar in 2025 about a 38-year-old man with serious injuries who was rescued in one of these hybrid rooms. Early TEG-guided injection of coagulation factors and angioembolization of the numerous bleeding vessels to control the hemorrhage in the retroperitoneal area helped the patient survive [141]. The importance of VETs and their prompt availability in emergency rooms for patients’ survival is also highlighted in a Chinese retrospective cohort study by Deng et al. (2018) [142] and in George et al. observational study (2022) [143].

Another study by Asim et al. from Qatar (2020–2021) reported that the high rates of blood transfusions and in-hospital death were correlated with hypofibrinogenemia and abnormal ROTEM FIBTEM results in 25% of their patients with serious injuries (*p* < 0.05) [47]. In line with Asim M et al., Ojuka et al. [144] also found that TIC was present at a notably high percentage (52.1%) in the 140 major trauma patients they assessed.

Over the past ten years, several trauma care centers worldwide prioritized the early diagnosis of coagulopathy by VETs [4,65,96,142,143,144,145]. The improvements in ER monitoring facilities in Qatar to meet the international accreditation (2014) improved in-hospital results [58]. In order to treat TBI, the American College of Surgeons (ACS) Committee on Trauma recommended in 2017 that trauma centers establish a dedicated intensive care unit (ICU). Adopting VET-guided guidelines for PBM was another recommendation [146,147]. In response, the Hamad Hospital in Qatar established a trauma intensive care unit (TICU) in 2016. Their experience was published by Chughtai et al. (2020) [138]. Earlier in 2014, a retrospective study indicated that Qatar’s post-trauma mortality rates are roughly 6–7% [148].

The Vision for 2030 of the Kingdom of Saudi Arabia aims to provide quality management and reduce the incidence of trauma [98]. In their 2020 literature analysis, Alharbi et al. used the Trauma System Maturity Index (TSMI) of the World Health Organization (WHO) and the American College of Surgeons (ACS) to analyze the peer-reviewed literature through a comprehensive database search [96]. They concluded that efforts are needed to develop a more robust national trauma system in Saudi Arabia, as it lacks several essential components, including PBM, compared with other systems worldwide. Saudi Arabia has one of the highest rates of trauma deaths from traffic accidents worldwide, with an average of 24.8 deaths per 100,000 inhabitants, according to the World Health Organization, 2015 [1,99,149].

In a retrospective cohort review, the King Abdulaziz Medical City trauma center in Riyadh investigated the use of TXA in 33% of 215 hemodynamically unstable trauma patients; however, TXA did not significantly reduce mortality. The importance of VETs as proof of fibrinolysis prior to administering TXA was not established and was one of the study’s limitations; 15.5% of patients who received TXA died in the hospital compared to 5.6% of those who did not [137,150,151,152]. Recently, VET-guided PBM has gradually replaced ratio-based transfusion in the Arabian Gulf region. The effects of employing the PBM program with VETs beyond trauma, in patients with severe obstetric hemorrhages of 2000 mL or more, were assessed in Ansari et al.’s 2026 retrospective observational study. They found that implementing PBM was linked to higher post-discharge hemoglobin values, decreased need of blood product transfusion, and significant cost savings of US$ 175,705 [153].

Further lessons can be learned from the management of military trauma from Afghanistan [80,153,154,155]. Here, Lammers et al. 2020 [81,154,155,156] demonstrated in a retrospective study including 3320 patients that VET guidance during initial resuscitation was independently associated with decreased mortality (OR, 0.63; *p* = 0.04) with an overall mortality after propensity analysis of 7.3% in the VET group versus 13.1% in the control group (*p* = 0.001). Accordingly, Lammers et al. suggested that it is time for a new standard in combat resuscitation.

## 5. Conclusions

VET consistently outperforms traditional coagulation tests in detecting early coagulation abnormalities and to predict massive transfusion and mortality in major trauma. Besides hyperfibrinolysis, fibrinogen deficiency often represents the first issue in TIC. Recent meta-analyses consistently demonstrated a significant reduction in mortality by implementing VET-guided bleeding management algorithms in severe trauma. Furthermore, venous thromboembolic events can be reduced, too.

The adoption of VET differs greatly throughout the Arabian Gulf council country (GCC) trauma systems. The variability in education, training, infrastructure, protocol implementation, and guidelines remains a challenge for widespread adoption. Improvement in outcome after severe trauma needs a culture change from just running massive transfusion protocols to massive hemorrhage protocols including VET-guided bleeding management as an essential part of PBM.

## Figures and Tables

**Table 1:** Characteristics of the included evidence.

Characteristic	Reports, n	% (n = 261)	Comment
2010–2014	51	19.5	Early period
2015–2019	90	34.5	Expansion of trauma-VET evidence
2020–2026	120	46.0	Most recent period
Randomized intervention	15	5.7	Randomized treatment/protocol comparison
Non-randomized/quasi-experimental	25	9.6	Treatment/protocol evaluation without randomization
Diagnostic/assay-performance	60	23.0	Diagnostic, threshold, or assay-comparison question
Prognostic factor/prediction	109	41.8	Outcome prediction or prognostic association
Observational phenotype/association	52	19.9	Phenotyping or other observational association

Data are reported as numbers (n) or percentage (%). For methodological purposes, categories are mutually exclusive and sum up to 261 reports.

**Table 2:** VET modalities and clinical evidence domains.

Domain or Modality	Reports, n	% of 261	Counting Rule
TEG	161	61.7	Overlapping modality count
ROTEM	105	40.2	Overlapping modality count
FIBTEM	45	17.2	ROTEM assay subset
EXTEM	38	14.6	ROTEM assay subset
INTEM	12	4.6	ROTEM assay subset
Coagulation/coagulopathy	215	82.4	Overlapping evidence domain
Diagnostic/prognostic performance	152	58.2	Overlapping evidence domain
Transfusion/blood products	152	58.2	Overlapping evidence domain
Mortality/survival	151	57.9	Overlapping evidence domain
Hemostatic management	135	51.7	Overlapping evidence domain
Fibrinogen	103	39.5	Overlapping evidence domain
Platelet function	98	37.5	Overlapping evidence domain
Fibrinolysis	74	28.4	Overlapping evidence domain
Thromboembolism	22	8.4	Overlapping evidence domain

Modality and clinical-domain counts are not mutually exclusive; one report could contribute to more than one category.

**Table 3:** Principal randomized evidence relevant to VET-guided hemostatic management.

Study	Population/Strategy	Main Result	Interpretive Boundary
Gonzalez et al. [57]	n = 111; TEG-guided MTP vs. conventional coagulation assays	28-day mortality 19.6% vs. 36.4%; *p* = 0.049; less early plasma/platelet use	Single-center pragmatic RCT
iTACTIC [58]	n = 396; VHA- vs. CCT-augmented major hemorrhage protocols	Alive/Free of massive transfusion at 24 h: 67% vs. 64%; OR 1.15 (95% CI 0.76–1.73); 28-day mortality 25% vs. 28%	No overall difference in primary or mortality outcomes
RETIC [59]	n = 100; ROTEM-defined coagulopathy; FFP vs. coagulation factor concentrates	Rescue therapy 52% vs. 4%; massive transfusion 30% vs. 12%; MOF 66% vs. 50%	Stopped early; open-label, single-center trial
FEISTY [60]	n = 100 randomized; 62 treated for hypofibrinogenemia; fibrinogen concentrate vs. cryoprecipitate	Median delivery time 29 vs. 60 min; *p* = 0.0001	Pilot trial; not powered for definitive patient-centered outcomes

CCT, conventional coagulation test; FFP, fresh frozen plasma; MOF, multiple organ failure; MTP, massive transfusion protocol; VHA, viscoelastic hemostatic assay.

**Table 4:** Studies demonstrating the superiority of viscoelastic hemostatic testing (VET)-guided bleeding management in severe trauma compared with CCT-guided bleeding management or the use of shock packs (transfusion-ratios).

First Author, Publication Year [Reference]	Study Type	VET Device (Number of Participants)	Impact on Mortality	Additional Findings
Riehl et al. 2022 [13]	Non-RCT (Retrospective Cohort Study)	ROTEM (1515 patients; 790 ROTEM; 725 controls); civilian trauma, Trauma Register DGU, TIC with risk of bleeding or need for blood transfusion before ICU	RR [95% CI) for in-hospital mortality, 0.86 [0.75; 0.99]	The use of ROTEM was associated with reduced mortality and an increased application of hemostatics and RBC transfusions.
Gonzalez et al. 2016 [57]	SC-RCT	TEG (111 trauma patients meeting the criteria for MTP activation; 56 TEG, 55 CCT); intention-to-treat analysis.	TEG group survival was significantly higher than the CCT group (log-rank *p* = 0.032, Wilcoxon *p* = 0.027); 20 deaths in the CCT group (36.4%) compared with 11 in the TEG group (19.6%) (*p* = 0.049). Most deaths occurred within the first 6 h from arrival (21.8% CCA group vs. 7.1% TEG group) (*p* = 0.032).	CCT patients required similar number of red blood cell units as the TEG patients [CCT: 5.0 (2–11), TEG: 4.5 (2–8)] *p* = 0.317), but more plasma units [CCT: 2.0 (0–4), TEG: 0.0 (0–3)] (*p* = 0.022), and more platelets units [CCT: 0.0 (0–1), TEG: 0.0 (0–0)] (*p* = 0.041) in the first 2 h of resuscitation.
Baksaas-Aasen et al. 2021 [58]	MC-RCT	TEG or ROTEM (396 trauma patients with MHP activation; 201 VET; 195 CCT); intention-to-treat analysis.	At 24 h, there was no difference in the proportion of patients who were alive and free of massive transfusion (VET: 67%, CCT: 64%, OR 1.15, 95% CI 0.76–1.73). 28-day mortality was not different overall (VET: 25%, CCT: 28%, OR 0.84, 95% CI 0.54–1.31), nor were there differences in other secondary outcomes or serious adverse events.	Only 29% (102/355) of patients were coagulopathic (PT-ratio > 1.2) at baseline. In the pre-specified subgroups of coagulopathic patients OR [95% CI] for 28-day mortality was 0.56 (0.26; 1.24), *p* = 0.070 and for patients with severe TBI OR [95% CI] was 0.28 [0.10; 0.74], *p* = 0.016.
Bugaev et al. 2020 [65]	MA	TEG or ROTEM (6 studies with 1748 participants)	RR [95% CI] for mortality, 0.75 [0.59; 0.95], *p* = 0.02, I2 = 0%	US Eastern Association for the Surgery of Trauma
Kvisselgaard et al. 2026 [66]	MA	TEG or ROTEM (19 RCTs with 2160 participants)	RR [95% CI] for mortality for all studies 0.76 [0.63; 0.92], I2 = 0%; RR [95% CI] for mortality for trials using TEG, 0.74 [0.57; 0.98], *p* = 0.03, I2 = 0%; RR [95% CI] for mortality for trials using ROTEM, 0.51 [0.32; 0.82], *p* = 0.005, I2 = 0%	RCTs only, multiple clinical settings, predominantly cardiac surgery (11 RCTs)
Tapia et al. 2013 [69]	Non-RCT (Retrospective Cohort Study)	TEG (289 patients; 165 TEG, 165 MTP); Civilian, MTP (≥6 RBCs/24 h), no TBI, age ≥ 15 years.	RR [95% CI] for 30-day mortality, 0.60 {0.39; 0.93]	Blunt trauma MTP patients who received ≥ 10 U RBC received more FFP (*p* = 0.02), with no change in mortality. Penetrating trauma patients who received ≥ 10 RBC received a similar volume of FFP; however, mortality increased from 54.1% for MTP versus 33.3% pre-MTP(TEG)-guided), *p* = 0.04.
Mitra et al. 2026 [77]	MA	TEG or ROTEM (29 studies with 11,006 participants)	RR [95% CI] for mortality, 0.789 [0.701; 0.888], *p* = 0.0003, I2 = 31%	Trauma only, RCTs and non-RCTs
Nardi et al. 2015 [78]	Non-RCT (Retrospective Cohort Study)	ROTEM (226 patients; 96 ROTEM; 130 Controls); adult civilian trauma, ≥3 RBCs/24 h.	RR [95% CI] for 28-day mortality, 0.68 [0.37; 1.25]	A 40% overall reduction in PRBCs was observed, together with a 65% reduction in plasma and a 52% reduction in platelets. Patients in the ROTEM group received fewer blood products: 6.51 units of PRBCs versus 8.14 units. Plasma transfusions decreased from 8.98 units to 4.21 units (*p* < 0.05), and platelets fell from 4.14 units to 2.53 units (*p* < 0.05).
Stein et al. 2017 [79]	Non-RCT (Retrospective Cohort Study)	ROTEM (731 patients; 408 ROTEM; 323 controls); civilian trauma, age ≥ 16 years.	RR [95% CI] for in-hospital mortality, 0.65 [0.51; 0.83]	Implementation of a revised trauma management strategy, which included ROTEM-guided coagulation manage-ment, was associated with a reduced incidence of massive transfusion and a reduction in transfusion of RBCs and FFP.
Unruh et al. 2019 [80]	Non-RCT (Retrospective Cohort Study)	TEG (67 patients; 47 TEG; 20 controls); adult civilian trauma with MTP activation	RR [95% CI] for mortality, 0.58 [0.33; 1.03]	Intent-to-treat analysis demonstrated a significant reduction in RBC transfusions (11 vs. 6 units, *p* = 0.001), number of patients receiving FFP (85.0 vs. 17.0%, *p* < 0.001), and platelets (75.0 vs. 38.3%, *p* = 0.006) in the TEG group.
Lammers et al. 2020 [81]	Non-RCT (Retrospective Cohort Study)	ROTEM (3320 patients; 594 ROTEM; 2726 controls); military, US DoD Trauma Registry, age ≥ 18 years.	RR [95% CI] for mortality, 0.69 [0.51; 0.94]	After adjusting for confounders, VET during initial resuscitation was independently associated with decreased mortality. Propensity analysis confirmed this survival advantage with a 57% reduction in overall mortality (7.3% vs. 13.1%; *p* = 0.001) for all patients requiring blood products.
Cochrane et al. 2020 [82]	Non-RCT (Prospec-tive Cohort Study)	TEG 6s (301 patients; 175 TEG; 126 CCTs; civilian trauma, age ≥ 16 years.	RR [95% CI] for 30-day mortality, 0.43 [0.25; 0.72]	TEG 6s-driven resuscitation algorithms were associated with reduced mortality, reduced blood product wastage, and are cost neutral compared to standard coagulation tests.
Pernod et al. 2021 [83]	Non-RCT (Retrospective Cohort Study)	ROTEM (244 patients; 122 ROTEM; 122 controls); civilian trauma with ≥4 RBCs within 24 h, age ≥ 18 years.	RR [95% CI] for 30-day mortality, 0.72 [0.51: 1.02]	After ROTEM implementation, fibrinogen was administered to more than 70% of patients and TXA to 100% of them. The observed mortality was lower than the predicted one, irrespective of FFP:RBC ratio.
Barquero Lopez et al. 2022 [84]	Non-RCT (Retrospective Cohort Study)	ROTEM sigma (^135 patients; 42 ROTEM; 93 controls); civilian trauma with ISS >15 and high risk of TIC, age ≥ 18 years.	RR [95% CI] for mortality, 0.74 [0.44; 1.23]	Progressive implementation of ROTEM resulted in increased use of fibrinogen concentrate over time (*p* < 0.001). Regression analysis shows that ROTEM group had a significant reduction in transfusion of RBCs (*p* = 0.005), FFP (*p* < 0.001), and platelets (*p* = 0.011). Regarding outcomes, ROTEM patients had less pneumonia (*p* = 0.019) and multiorgan failure (*p* < 0.001).
Beyersdorf et al. 2024. [85]	Non-RCT (Case-Control Study)	ROTEM (3444 patients; 1722 ROTEM; 1722 controls); civilian trauma; Trauma Register DGU, estimated risk of massive transfusion > 2% based on TASH score, age ≥ 16 years.	RR [95% CI] for mortality, 0.84 [0.77; 0.93]	Early thromboelastometry can lead to a more targeted coagulation management but is not yet widely available.
Mitra et al. 2024 [86]	Non-RCT (Prospec-tive Cohort Study)	ROTEM (192 patients; 40 ROTEM; 152 controls); ANZTR, civilian shocked trauma patients (SBP ≤ 90 mmHg) from two trauma centers, formulaic vs. VET approach, age ≥ 18 years.	RR [95% CI] for in-hospital mortality, 0.49 [0.18; 1.31]	Formulaic resuscitation was associated with significantly more blood components transfused (adjusted incidence rate ratio [95% CI], 1.5; [1.4–1.7], *p* < 0.001). Using a formulaic approach, patients have been administered more RBCs, FFP, and platelets, but fewer cryoprecipitate.
Salehi et al. 2023 [87]	Non-RCT (Retrospective Cohort Study)	ROTEM (64 patients; 43 ROTEM; 21 controls); civilian trauma, Level 1 trauma center with MTP activation, mean age 42 years.	RR [95% CI] for in-hospital mortality, 0.63 [0.27; 1.45]	The use of fibrinogen concentrate was significantly higher in the ROTEM-period group (pre-ROTEM mean 0.2 vs. ROTEM-period mean 0.8; *p* = 0.006). There was no significant difference in the number of units of RBCs, platelets, cryoprecipitate, or FFP transfused between these groups.
Liu et al. 2024 [88]	Non-RCT (Retrospective Cohort Study)	ROTEM (97 patients; 31 ROTEM, 66 controls); civilian trauma, ≥10 RBCs within 24 h or ≥4 RBC within 4 h, age ≥ 18 years.	RR [95% CI] for in-hospital mortality, 0.64 [0.29; 1.43]	In per-protocol-analysis, the 31/97 (32%) with ROTEM-guided transfusion used less RBCs (median [IQR]: 6 [6,7,8] vs. 8 [6,7,8,9,10,11,12] U, *p* = 0.03) than patients whose transfusion was not ROTEM-guided. They were also less likely to receive FFP (2/31 [6%] vs. 45/66 [68%], *p* < 0.0001) or platelets (2/31 [6%] vs. 31/66 [47%], *p* < 0.0001); they were, however, more likely to receive fibrinogen products (26/31 [84%] vs. 38/66 [58%], *p* = 0.01).

Australian and New Zealand Trauma Registry (ANZTR), Confidence Interval (CI), conventional coagulation tests (CCTs), Deutsche Gesellschaft für Unfallchirurgie (GGU), fresh frozen plasma (FFP), Injury Severity Score (ISS), hours (h), intensive care unit (ICU), interquartile range (IQR), meta-analysis (MA), massive hemorrhage protocol (MHP), massive transfusion protocol (MTP), multicenter (MC), non-RCT (cohort study or case–control study), Randomized Controlled Trial (RCT), rotational thromboelastometry (ROTEM), red blood cells (RBCs), risk ratio (RR), Systolic Blood Pressure (SBP), single-center (SC), trauma-induced coagulopathy (TIC), traumatic brain injury (TBI), thromboelastography (TEG), units (U), viscoelastic testing (VET).

## Data Availability

Data are available from the authors upon reasonable request.
